# Spatial reversal learning defect coincides with hypersynchronous telencephalic BOLD functional connectivity in APP^NL-F/NL-F^ knock-in mice

**DOI:** 10.1038/s41598-018-24657-9

**Published:** 2018-04-19

**Authors:** Disha Shah, Amira Latif-Hernandez, Bart De Strooper, Takashi Saito, Takaomi Saido, Marleen Verhoye, Rudi D’Hooge, Annemie Van der Linden

**Affiliations:** 1 Bio-Imaging Lab, University of Antwerp, Antwerpen, Belgium; 2 Laboratory of Biological Psychology, KU Leuven, Leuven, Belgium; 3 VIB Center for Brain and Disease Research, KU Leuven, Leuven, Belgium; 4 Laboratory for Proteolytic Neuroscience, Riken Brain Science Institute, Saitama, Japan

## Abstract

Amyloid pathology occurs early in Alzheimer’s disease (AD), and has therefore been the focus of numerous studies. Transgenic mouse models have been instrumental to study amyloidosis, but observations might have been confounded by APP-overexpression artifacts. The current study investigated early functional defects in an APP knock-in mouse model, which allows assessing the effects of pathological amyloid-beta (Aβ) without interference of APP-artifacts. Female APP^NL/NL^ knock-in mice of 3 and 7 months old were compared to age-matched APP^NL-F/NL-F^ mice with increased Aβ42/40 ratio and initial Aβ-plaque deposition around 6 months of age. Spatial learning was examined using a Morris water maze protocol consisting of acquisition and reversal trials interleaved with reference memory tests. Functional connectivity (FC) of brain networks was assessed using resting-state functional MRI (rsfMRI). The Morris water maze data revealed that 3 months old APP^NL-F/NL-F^ mice were unable to reach the same reference memory proficiency as APP^NL/NL^ mice after reversal training. This cognitive defect in 3-month-old APP^NL-F/NL-F^ mice coincided with hypersynchronous FC of the hippocampal, cingulate, caudate-putamen, and default-mode-like networks. The occurrence of these defects in APP^NL-F/NL-F^ mice demonstrates that cognitive flexibility and synchronicity of telencephalic activity are specifically altered by early Aβ pathology without changes in APP neurochemistry.

## Introduction

Alzheimer’s disease (AD) is a neurodegenerative disorder, characterized by progressive impairments in learning and memory, and other cognitive dysfunctions^1^. Its pathological hallmarks include the accumulation of extracellular amyloid plaques and intracellular tau tangles, but the mechanisms leading to functional defects and full-blown AD pathology are poorly understood. Available treatment offers symptomatic benefit without halting or reversing disease progression. AD pathology progresses over decades before symptoms develop, at which stage the damage might be too extensive, emphasizing the importance of early diagnosis and intervention. In the interest of establishing early biomarkers and therapy, many studies focused on the pathological changes at initial stages of the disease. According to the amyloid cascade hypothesis, accumulation of amyloid-beta (Aβ) peptide is such an event that impairs brain function early on, and triggers tau pathology and neurodegeneration^2^. Recent studies suggest that early disease signs are not caused by Aβ plaque deposition as such, but rather by pre-plaque levels of soluble Aβ peptides with high Aβ42/40 ratio^3–5^.

Transgenic mouse models that overexpress APP have been instrumental to our present knowledge of AD pathogenesis in general, and Aβ-related mechanisms in particular. Much work on transgenic mouse models has, however, been confounded by the possibility that overexpression of APP and APP fragments induces artificial phenotypes. For example, overexpression of wild-type APP can interfere with cellular transport mechanisms, cause loss of synapses, and lead to memory disruption without actual Aβ involvement^6,7^. Knock-in mouse models, which express APP at wild-type levels while overproducing pathogenic Aβ, have been specifically developed to control for these possible confounds^6,7^. We will presently use such a knock-in model to investigate the putative occurrence of functional defects at the pre-plaque stage. High Aβ42/40 ratio prior to plaque deposition has been suggested to cause synaptic and neural network dysfunction leading to cognitive defects in early phases of AD^3–5^.

In the current study, we compared APP^NL-F/NL-F^ knock-in mice with high Aβ42/40 ratio to APP^NL/NL^ mice at two time points that reflect early pathological stages, i.e. before and at the initial stage of plaque deposition^8^. Different aspects of spatial learning and memory were assessed using an extended protocol in the Morris water maze task to model declarative-like memory functions and response-flexibility or working memory^9^. Functional connectivity (FC) between telencephalic regions was studied using non-invasive resting-state functional Magnetic Resonance Imaging (rsfMRI), which uses low frequency (0.01–0.1 Hz) fluctuations in blood-oxygenation-level-dependent (BOLD) signals to measure fluctuations of neuronal activity. FC is defined as the temporal correlation of BOLD fluctuations between spatially distinct brain regions^10,11^. Considering that rsfMRI has been applied extensively in AD patients, it allows relatively easy translation to the clinic^12^. Previous studies have demonstrated the usefulness of rsfMRI to assess the functionality of brain networks in AD-related pharmacological models and transgenic mouse models^13–16^.

## Results

### Spatial learning in the Morris water maze

**Figure 1:**
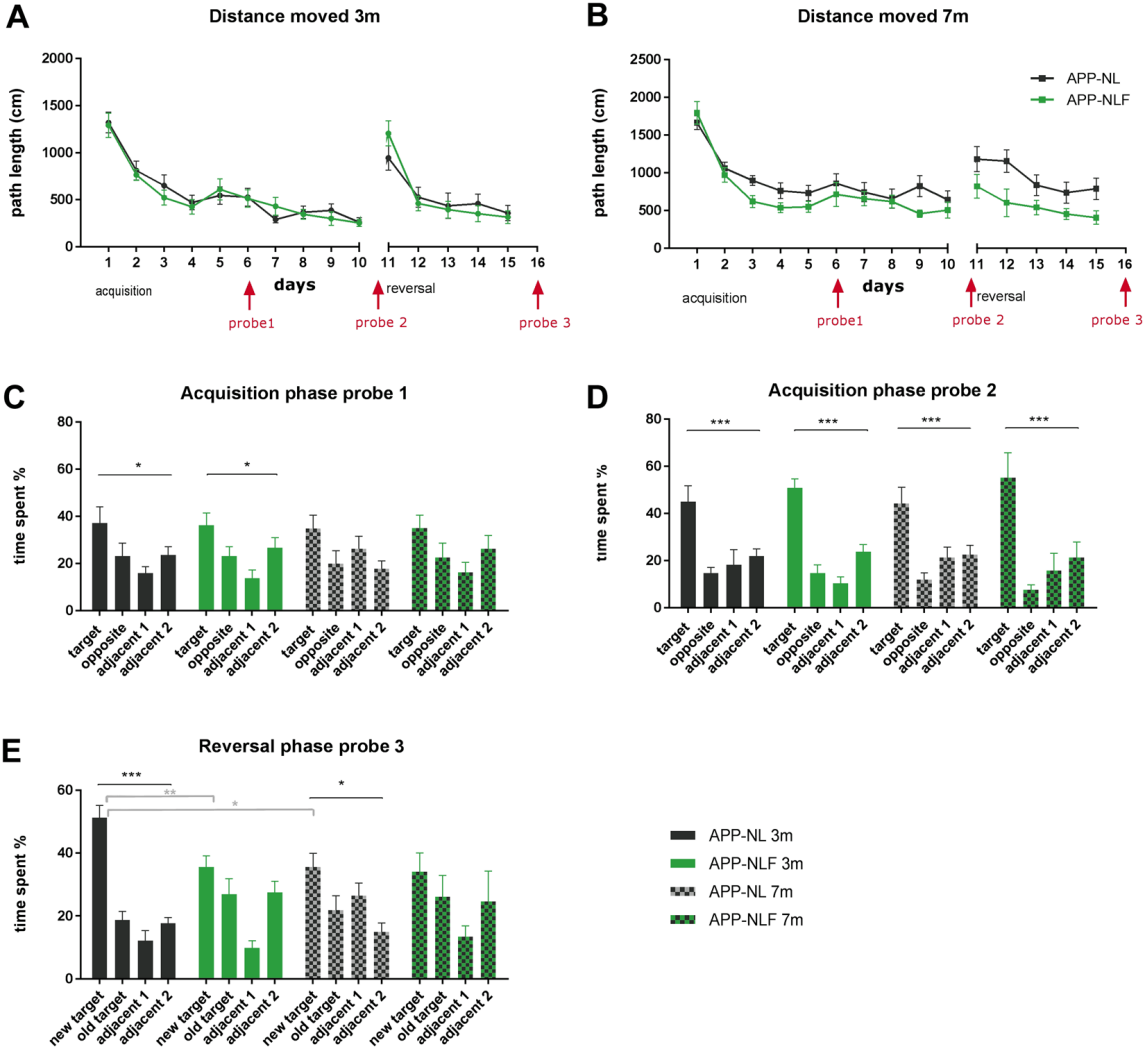
Spatial learning in the Morris water maze. (**A**,**B**) Show learning curves of the acquisition (10 days) and reversal phase (5 days) as distance moved (cm) for APP^NL/NL^ and APP^NL-F/NL-F^ mice at 3 months (**A**) and 7 months (**B**) of age. Timing of the probe trials are indicated in red i.e. on day 6 (acquisition probe 1), day 11 (acquisition probe 2) and day 16 (reversal probe 3). (**C**–**E**) Show the results of the probe trials as % time spent in each quadrant for APP^NL/NL^ and APP^NL-F/NL-F^ mice at 3 and 7 months of age during the acquisition (**C**,**D**) and reversal phase (**E**). *p < 0.05, **p < 0.01, ***p < 0.001, corrected for multiple comparisons, one-way ANOVA = black, two-way ANOVA = grey.

At 3 months of age (Fig. 1), repeated measures (RM) two-way ANOVA showed no statistical difference between the learning curves of APP^NL-F/NL-F^ and APP^NL/NL^ mice during acquisition (RM-ANOVA, ‘genotype x time’ interaction F_9,135_ = 0.446, p = 0.907, genotype effect F_1,15_ = 0.094, p = 0.763) or reversal trials (RM-ANOVA, ‘genotype x time’ interaction F _4,60_ = 1.399, p = 0.245, genotype effect F_1,15_ = 1.244*10^−5^, p = 0.997). RM-ANOVA indicated that all animals learnt the location of the platform during acquisition (time effect F_9,135_ = 29.86, p < 0.0001) and reversal learning (time effect F_4,60_ = 23.36, p < 0.0001). There was no significant difference in velocity between groups during the acquisition (‘genotype x time’ interaction F_9,135_ = 1.416, p = 0.187, genotype effect F_1,15_ = 0.066, p = 0.800) or reversal phase (‘genotype x time’ interaction F_4,60_ = 1.567, p = 0.194, genotype effect F_1,15_ = 0.611, p = 0.446) (Figure S1). Probe trials were conducted on days 6 (acquisition probe 1) and 11 (acquisition probe 2) to assess spatial reference memory. The probe trials of the acquisition phase showed a significant preference for the target quadrant compared to the other quadrants for both APP^NL/NL^ and APP^NL-F/NL-F^ mice (one-way ANOVA, probe 1 APP^NL/NL^ p = 0.037; probe 1 APP^NL-F/NL-F^ p = 0.01; probe 2 APP^NL/NL^ p = 0.0007; probe 2 APP^NL-F/NL-F^, p < 0.0001), but no group differences were observed (two-way ANOVA, probe 1 “genotype x time in quadrant” interaction effect, F_3,60_ = 0.1184, p = 0.948; probe 2 interaction effect, F_3,60_ = 0.956, p = 0.419). A third probe trial was conducted on day 16 (i.e., after 5 days of reversal training). During this trial, APP^NL/NL^ mice did show a preference for the new target quadrant (one-way ANOVA p < 0.0001), whereas APP^NL-F/NL-F^ mice did not (one-way ANOVA p = 0.234). A significant group difference in preference for the new target quadrant was observed during the probe test of the reversal phase (two-way ANOVA, ‘genotype x time in quadrant’ interaction effect, F_3,60_ = 6.123, p = 0.001, post-hoc Sidak test preference for new target quadrant p = 0.0064).

At 7 months of age (Fig. 1), there was no statistical difference in the learning curve of the acquisition phase (RM-ANOVA ‘genotype x time’ interaction F_9,135_ = 0.523, p = 0.854; genotype effect F_1,1986_.817, p = 0.186;) or reversal phase (RM-ANOVA, ‘genotype x time’ interaction F_4,60_ = 0.638, p = 0.637, genotype effect F_1,15_ = 3.224, p = 0.095) between APP^NL-F/NL-F^ and APP^NL/NL^ mice. RM-ANOVA indicated that all animals learnt the location of the platform during acquisition (time effect F_9,135_ = 9.266, p < 0.0001) and reversal learning (time effect F_4,60_ = 3.486, p = 0.0123). There was no significant difference in velocity between groups during the acquisition phase (RM-ANOVA, ‘genotype x time’ interaction F_9,135_ = 1.393, p = 0.199, genotype effect F_1,15_ = 0.006, p = 0.935) or reversal phase (‘genotype x time’ interaction F_4,60_ = 0.562, p = 0.691, genotype effect F_1,15_ = 2.698, p = 0.121) (Figure S1). The first probe trial of the acquisition phase showed no significant preference for the target quadrant compared to the other quadrants for both APP^NL/NL^ (one-way ANOVA p = 0.09) and APP^NL-F/NL-F^ mice (one-way ANOVA p = 0.1344) and no group differences were observed (two-way ANOVA, ‘genotype x time in quadrant’ interaction effect, F_3,60_ = 1.098, p = 0.360). The second probe trial of the acquisition phase showed a significant preference for the target quadrant in both APP^NL/NL^ (one-way ANOVA p = 0.0003) and APP^NL-F/NL-F^ mice (one-way ANOVA p = 0.0009), but no group differences were observed (two-way ANOVA, genotype x time in quadrant’ interaction effect, F_3,60_ = 0.818, p = 0.483). The third probe trial (i.e., after 5 days of reversal training) demonstrated that the APP^NL/NL^ control mice did show a slight preference for the new target quadrant (one-way ANOVA p = 0.01), whereas the APP^NL-F/NL-F^ mice did not (one-way ANOVA, p = 0.224). However, no group difference in preference for the new target quadrant was observed during the reversal probe test (two-way ANOVA, ‘genotype x time in quadrant’ interaction effect, F_3,60_ = 1.754, p = 0.165).

### Functional connectivity within brain networks

**Figure 2:**
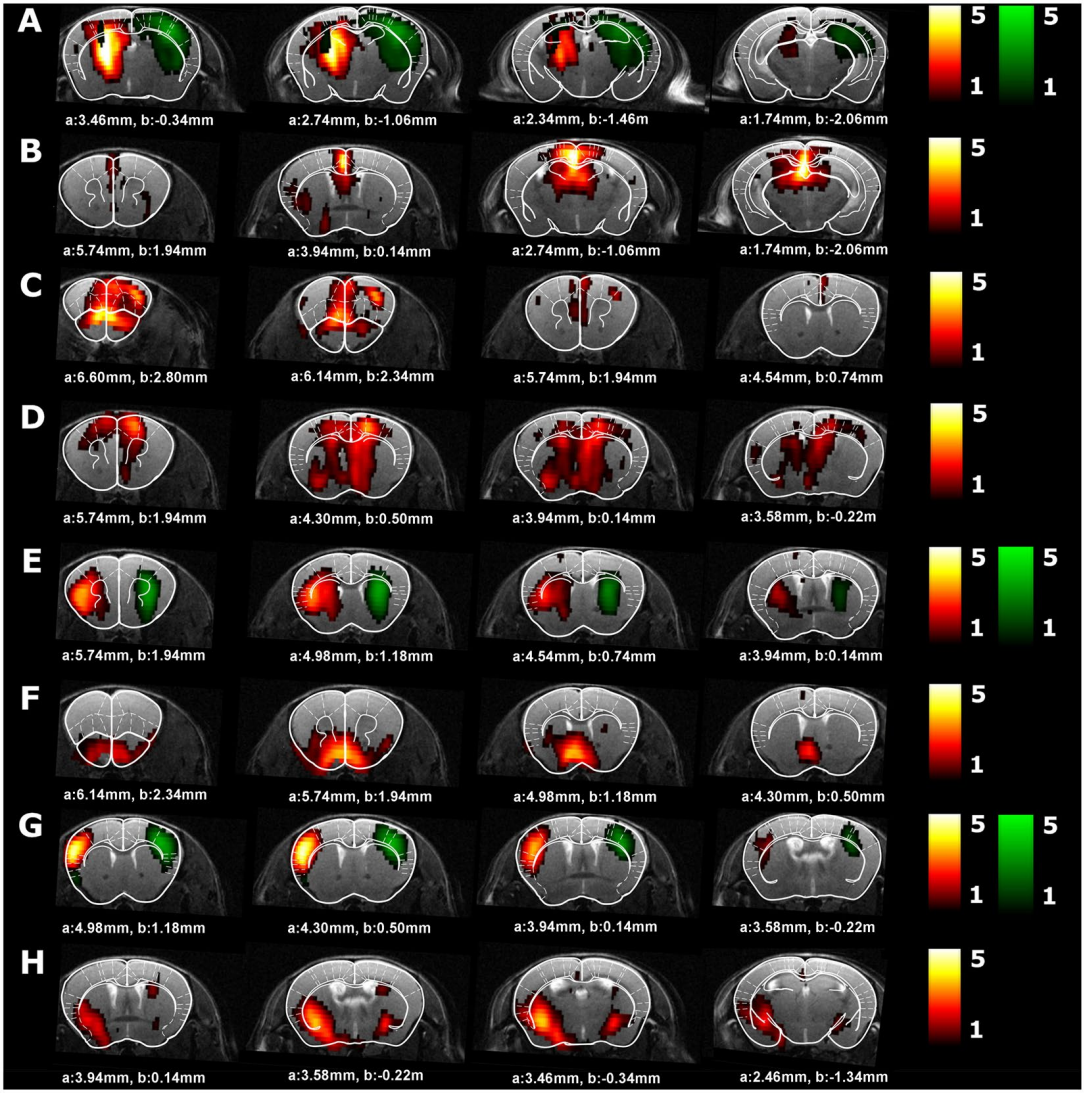
ICA components in APP^NL/NL^ mice. This figure shows which neurologically relevant ICA components were observed in APP^NL/NL^ mice. Four slices of the ICA components are shown on an anatomical T2-weighted MRI image and overlaid with the Franklin and Paxinos anatomical mouse brain atlas^29^ with indication of the stereotactic coordinates a = interaural, b = bregma. The color scale represents the z-score i.e. the strength of FC within each ICA component. Homologous ICA networks are shown on the same image (left ICA component in red scale, right ICA component in regreen scale). (**A**) hippocampus network, (**B**) Default-mode like (DMN-like) network, (**C**) frontal/cingulate network, (**D**) cingulate/thalamus network, (**E**) caudate putamen network, (**F**) nucleus accumbens/hypothalamus network, (**G**) sensorimotor network, (**H**) piriform network.

Figure 2 shows the neurologically relevant ICA components, each of which consist of voxels that show highly correlated BOLD time courses, and therefore form resting-state networks. The following networks were identified: the hippocampal network, default-mode-like (DMN) network, the frontal/cingulate network, the cingulate/thalamus network, the caudate putamen network, the nucleus accumbens/hypothalamus network, the sensorimotor network and the piriform network.

**Figure 3:**
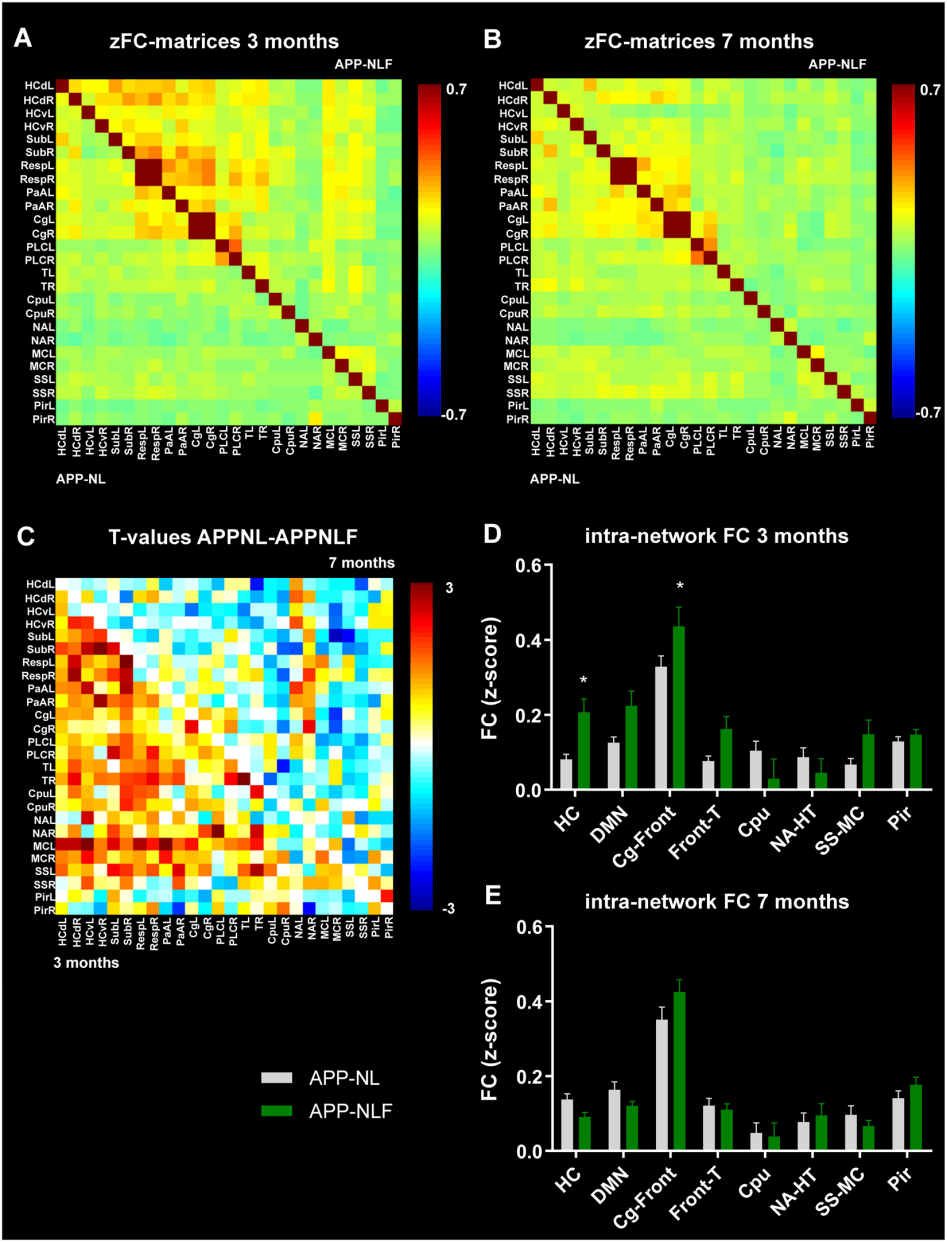
BOLD FC within brain networks. (**A**,**B**) zFC-matrices of 3 months (**A**) and 7 months (**B**) old APP^NL/NL^ (lower half of the matrix) and APP^NL-F/NL-F^ mice (upper half of the matrix). Color scale represents the z-score i.e. strength of FC between each pair of brain regions. (**C**) T-values representing the statistical group difference (two-sample T-test) between APP^NL/NL^ and APP^NL-F/NL-F^ mice at 3 months (lower half of the matrix) and 7 months of age (upper half of the matrix). Color scale represents the T-values. (**D**,**E**) graph shows FC within each brain network in 3 months (**D**) and 7 months (**E**) old APP^NL/NL^ and APP^NL-F/NL-F^ mice. *p < 0.05, **p < 0.01, ***p < 0.001, two-way-ANOVA. Abbreviations are listed in Table 1, L = left hemisphere, R = right hemisphere.

Table 1 specifies the brain regions observed in each of these ICA components. Those brain regions were used to compute FC-matrices (Fig. 3). At 3 months of age there was an overall hypersynchrony of BOLD FC in APP^NL-F/NL-F^ mice compared to APP^NL/NL^ mice (Fig. 3A), as is shown by the T-values representing the difference between groups (Fig. 3C). BOLD FC within brain networks was significantly increased in APP^NL-F/NL-F^ mice in the hippocampal (two-way ANOVA, p = 0.01) and frontal/cingulate networks (two-way ANOVA, p = 0.03) (Fig. 3D). At 7 months there is an overall hyposynchrony of BOLD FC in APP^NL-F/NL-F^ mice compared to APP^NL/NL^ mice (Fig. 3B), as is shown by the T-values representing the difference between groups (Fig. 3C). However, the decrease of BOLD FC within brain networks observed in APP^NL-F/NL-F^ vs. APP^NL/NL^ mice did not reach statistical significance when correcting for multiple comparisons (Fig. 3E).

**Table 1:** Brain regions in the ICA components.

ICA component/brain network	Brain regions	Abbreviation
Hippocampus	subiculum dorsal hippocampus ventral hippocampus	Sub dHC vHC
DMN-like	Prefrontal cortex Cingulate cortex Retrosplenial cortex Hippocampus Thalamus Parietal association cortex	PLC Cg Resp HC T PaA
Frontal/cingulate	Prefrontal cortex Cingulate cortex	PLC Cg
Cingulate/thalamus	Cingulate cortex Thalamus	Cg T
Caudate putamen	Caudate putamen	Cpu
Nucleus accumbens/hypothalamus	Nucleus accumbens Anterior hypothalamus	NA HT
Sensorimotor	Somatosensory cortex Motor cortex	SS MC
Piriform	Piriform cortex	Pir

### Connectivity between brain networks

**Figure 4:**
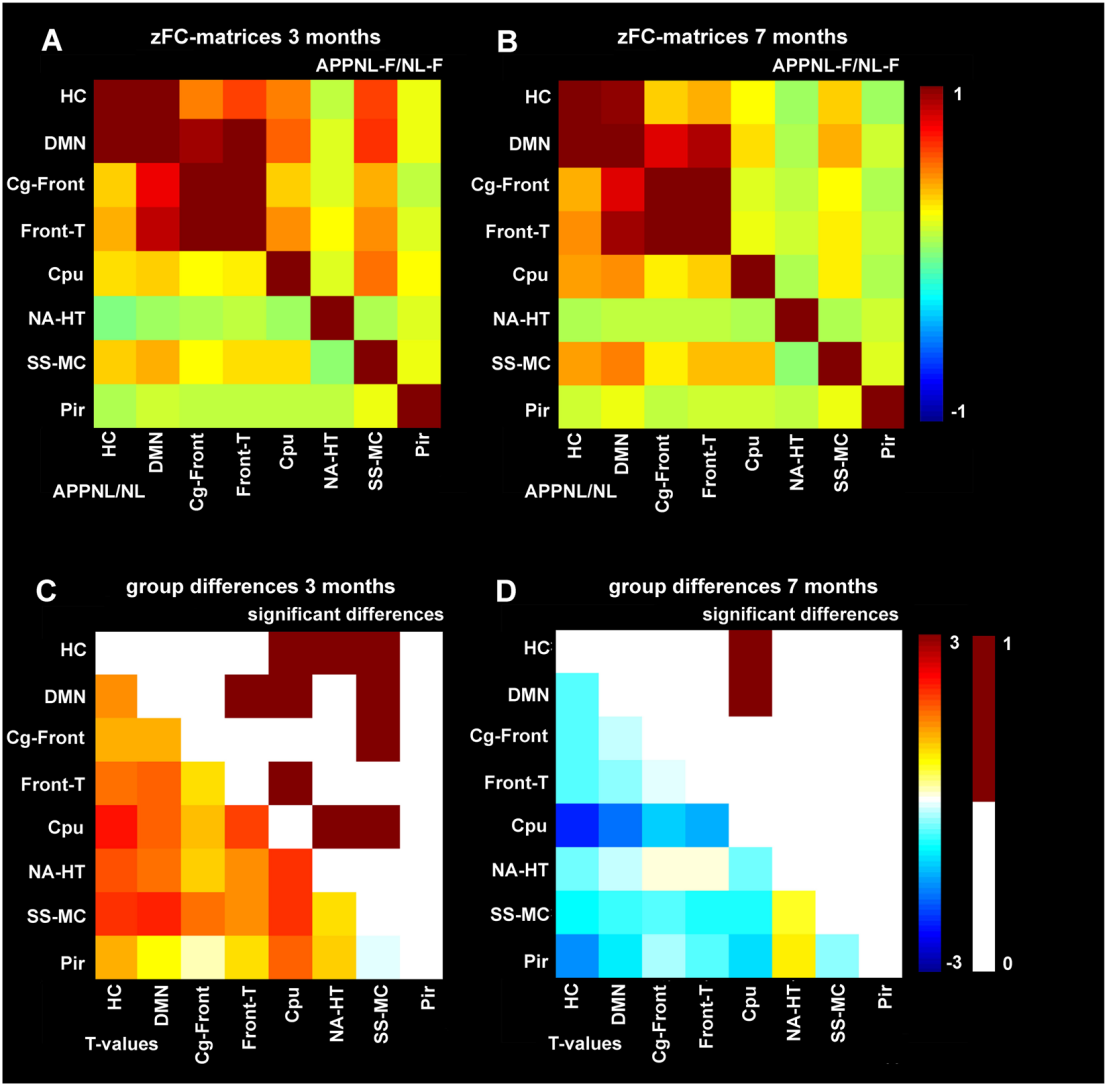
BOLD FC within between networks. (**A**,**B**) zFC-matrices of 3 months (**A**) and 7 months (**B**) old APP^NL/NL^ (lower half of the matrix) and APP^NL-F/NL-F^ mice (upper half of the matrix). Color scale represents the z-score i.e. strength of FC between each pair of brain networks. (**C**,**D**) T-values representing the statistical group difference (lower half of the matrix) and binary matrix with statistically significant differences (upper half of the matrix) between APP^NL/NL^ and APP^NL-F/NL-F^ mice at 3 months (**C**) and 7 months of age (**D**). Color scale represents the T-values (twog-sample T-test). Abbreviations are listed in Table 1.

Besides analyzing BOLD FC *within* networks, FC *between* brain networks resulting from ICA was additionally assessed. At 3 months of age, significant hypersynchrony of BOLD FC was observed in APP^NL-F/NL-F^ vs. APP^NL/NL^ mice (two sample T-test of zFC-matrices) between the hippocampus-caudate putamen (p = 0.01), hippocampus-nucleus accumbens (p = 0.02), hippocampus-sensorimotor (p = 0.01), DMN like-frontal/thalamus (p = 0.03), DMN like-caudate putamen (p = 0.03), DMN like-sensorimotor (p = 0.01), cingulate/frontal-sensorimotor (p = 0.03), frontal/thalamus-caudate putamen (p = 0.02), caudate putamen-nucleus accumbens (p = 0.01) and caudate putamen-sensorimotor (p = 0.01) (Fig. 4). At 7 months of age, significant hyposynchrony of BOLD FC was observed in APP^NL-F/NL-F^ vs. APP^NL/NL^ mice (two sample T-test of zFC-matrices) between the hippocampus-caudate putamen (p = 0.008) and DMN like- caudate putamen (p = 0.03) (Fig. 4).

**Figure 5:**
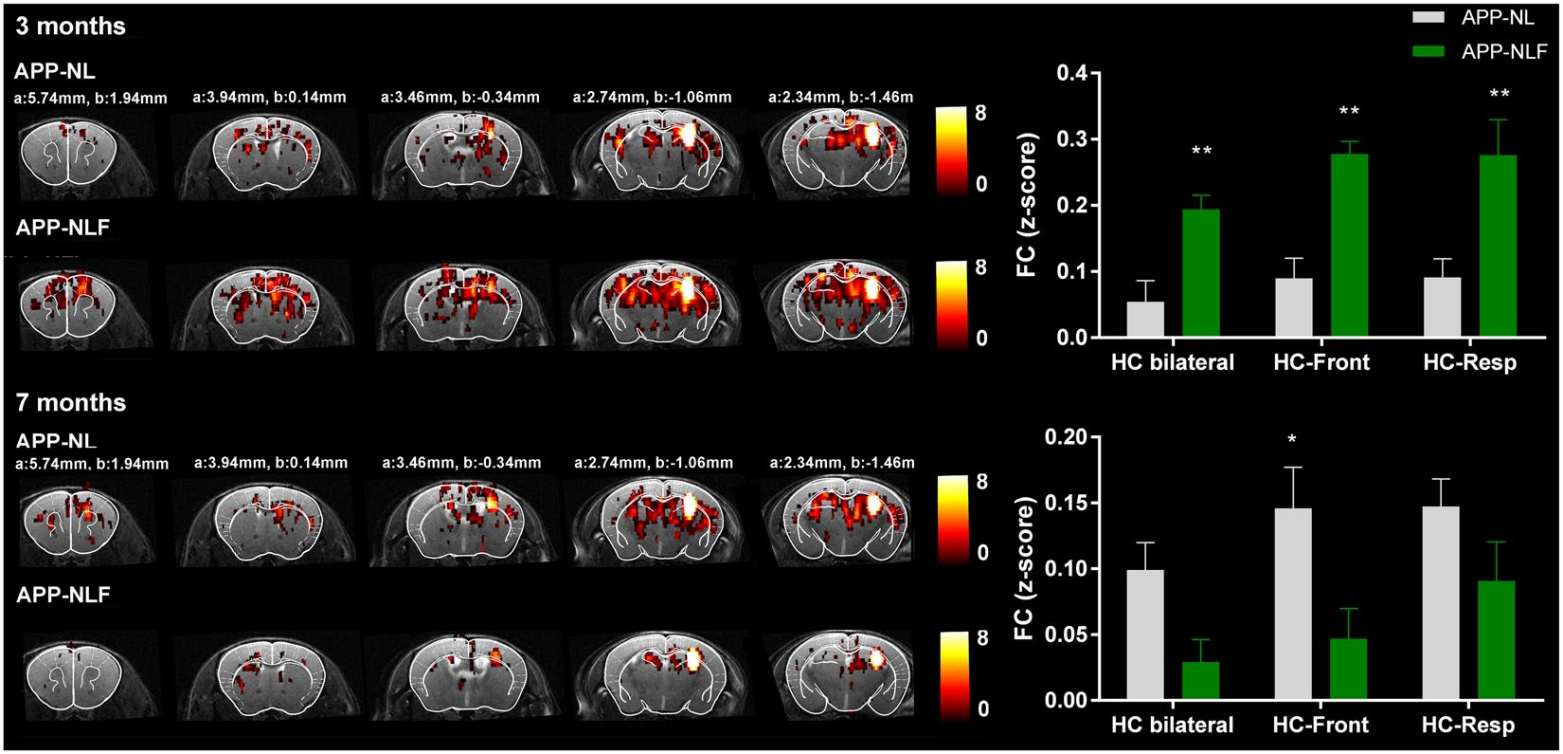
FC-map of the hippocampus. Statistical zFC-maps of the right hippocampus are shown for 3 and 7 months old APP^NL/NL^ and APP^NL-F/NL-F^ mice. Five slices of the zFC-maps are shown an anatomical T2-weighted MRI image and overlaid with the Franklin and Paxinos anatomical mouse brain atlas^29^ with indication of the stereotactic coordinates a = interaural, b = bregma. Color scale represents the T-value (one-sample T-test), i.e. strength of FC of the right hippocampus with all other voxels in the brain. Graphs show strength of FC as z-scores for FC of the hippocampus bilaterally, FC between the hippocampus and frontal cortex and between the hippocampus and retrosplenial cortex. *p < 0.05, **p < 0.01, ***p < 0.001, two-way ANOVA, corrected for multiple comparisons.

Compared to APP^NL/NL^ mice, APP^NL-F/NL-F^ mice showed deficits of cognitive flexibility observed during reversal learning in the MWM task. These type of cognitive functions depend on the functionality of the hippocampus and its connection to the retrosplenial areas and frontal cortex. Additionally, the analyses of FC within and between brain networks showed a significant impairment of hippocampal FC in 3 months old APP^NL-F/NL-F^ mice. Therefore, to have a more detailed view of FC of the hippocampus with other brain regions, FC-maps of the right hippocampus were computed for each group (Fig. 5). The hippocampal FC map shows that at 3 months of age the APP^NL-F/NL-F^ mice demonstrated hypersynchronous BOLD FC in the hippocampus bilaterally (two-way ANOVA, p = 0.007) compared to APP^NL/NL^ mice. Additionally, hypersynchronous BOLD FC between the hippocampus and the frontal cortex (two-way ANOVA, p = 0.001) and between the hippocampus and the retrosplenial cortex (two-way ANOVA, p = 0.001) was also observed in APP^NL-F/NL-F^ vs. APP^NL/NL^ mice. At 7 months of age, APP^NL-F/NL-F^ mice showed no significant hyposynchrony of BOLD FC between the hippocampus bilaterally (two-way ANOVA, p = 0.111) or between the hippocampus and retrosplenial cortex (two-way ANOVA, p = 0.118) compared to APP^NL/NL^ mice. Notably, APP^NL-F/NL-F^ mice showed significant hyposynchrony of BOLD FC between hippocampus and frontal cortex (two-way ANOVA, p = 0.02).

**Figure 6:**
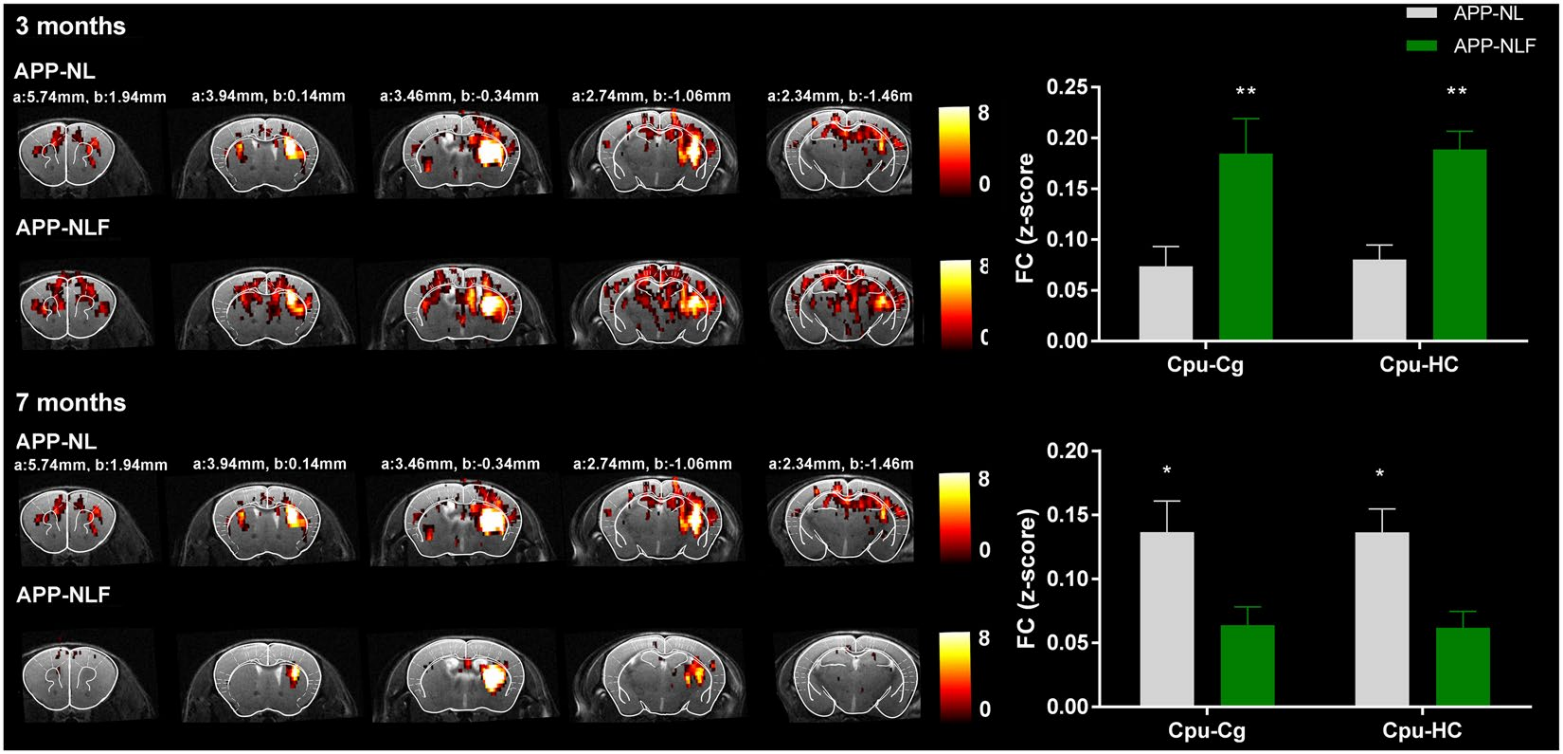
FC-map of the caudate putamen. Statistical zFC-maps of the right caudate putamen are shown for 3 and 7 months old APP^NL/NL^ and APP^NL-F/NL-F^ mice. Five slices of the zFC-maps are shown an anatomical T2-weighted MRI image and overlaid with the Franklin and Paxinos anatomical mouse brain atlas^29^ with indication of the stereotactic coordinates a = interaural, b = bregma. Color scale represents the T-value (one-sample T-test), i.e. strength of FC of the right caudate putamen with all other voxels in the brain. Graphs show strength of FC as z-scores for FC between the caudate putamen and cingulate regions, and between the caudate putamen and hippocampus. *p < 0.05, **p < 0.01, ***p < 0.001, two-way ANOVA, corrected for multiple comparisons.

Moreover, the analyses of FC between brain networks showed a significant impairment of FC between the striatal (caudate putamen) and DMN-like network, and between the caudate putamen and hippocampus, in APP^NL-F/NL-F^ mice at 3 months, but also at 7 months of age. These findings were confirmed when analyzing the FC maps of the right caudate putamen for each group (Fig. 6). The caudate putamen FC map shows hypersynchrony of BOLD FC between the caudate putamen and cingulate region, which is a major node of the DMN-like network (two-way ANOVA, p = 0.001), as well as between the caudate putamen and hippocampus (two-way ANOVA, p = 0.001) at 3 months of age in the APP^NL-F/NL-F^ vs. APP^NL/NL^ mice. At 7 months of age, APP^NL-F/NL-F^ mice showed a significant hyposynchrony of BOLD FC between caudate putamen and cingulate regions (two-way ANOVA, p = 0.02), as well as between the caudate putamen and hippocampus (two-way ANOVA, p = 0.02) compared to APP^NL/NL^ mice.

## Discussion

The current study aimed at investigating functional changes associated with early Aβ pathology in an APP knock-in mouse model, which allows assessing these effects without confounds by APP or APP fragments. APP^NL-F/NL-F^ mice were constructed to display pathologically increased Aβ42/40 ratio compared to APP^NL/NL^ mice^8^. In the present report, we examined these mice well before (3 months) and at the initial stages of Aβ plaque deposition (7 months) (Figure S2). Similar to brain pathology in AD patients, plaques in the cortex of APP^NL-F/NL-F^ mice consist mainly of the Aβ42 species^8^. APP^NL-F/NL-F^ mice (but not APP^NL/NL^ mice) display initial Aβ plaques around 6 months of age, concurrent with accumulation of microglia and astrocytes, whereas synaptic loss was reported to occur not before 9–12 months of age^8^.

Behavior defects were previously shown to occur very late in these mice. Using APP^NL/NL^ mice as controls, impaired avoidance behavior and compulsivity were observed in APP^NL-F/NL-F^ mice at 8–12 months, and deficits in place preference learning between 13–17 months^17^. APP^NL-F/NL-F^ mice also showed deficits in Y-maze alteration at 18 months of age^8^. In accordance, we failed to observe differences in 3- and 7-month-old APP^NL/NL^ and APP^NL-F/NL-F^ mice during the acquisition phase of MWM learning. However, we did find indications of impaired spatial reversal learning in 3-month-old APP^NL-F/NL-F^ mice. After a series of reversal learning trials, during which again no major impairment was observed, APP^NL-F/NL-F^ mice failed to show similar reference memory proficiency in the reversal probe trial compared to APP^NL/NL^ mice. Apparently, general learning abilities were not affected in these mice, but they failed to acquire and/or remember the novel platform position as effectively as APP^NL/NL^ mice. At 7 months of age, again no differences were observed during acquisition and reversal trails between APP^NL-F/NL-F^ and APP^NL/NL^ mice. During the reversal trials APP^NL-F/NL-F^ mice seem to travel less distance than APP^NL/NL^ mice, suggesting improved performance. However, this difference was not statistically significant and the reversal probe trial showed that 7-month-old APP^NL-F/NL-F^ mice were actually slightly less accurate in searching for the platform than APP^NL/NL^ mice of that age. We also observed that APP^NL/NL^ mice failed to acquire the same spatial proficiency after reversal training as they did at 3 months of age, which might have been due to age-related deteriorations in cognitive flexibility. Indeed, APP^NL/NL^ mice reportedly show age-dependent defects of learning abilities which could overshadow group differences^17,18^. As a probable result, the difference between APP^NL-F/NL-F^ and APP^NL/NL^ mice in the reversal probe trial was much less pronounced than that at 3 months.

Thus, impaired cognitive flexibility appears to be the earliest behavioral or cognitive change in this mouse model. The reversal defect in 3-month-old APP^NL-F/NL-F^ mice is especially interesting since it occurred at an age when these mice hardly have Aβ plaques or other signs of major neuropathology. Notably, our rsfMRI data revealed hypersynchrony between telencephalic neural networks at this specific age, in particular within hippocampus and between hippocampus and prefrontal cortex. Reversal learning requires animals to update a previously learnt associative spatial map. Deficits in reversal learning indicate decreased adaptability of acquired information to changing environmental demands (i.e., cognitive flexibility). It has been well established that different aspects of spatial learning in the MWM task depend on telencephalic regions, most prominently hippocampus^9,19,20^. However, reversal learning abilities appear to depend specifically on a network of reciprocal connections and subsequent crosstalk between hippocampus, prefrontal cortex and striatum^9,21,22^.

RsfMRI is a non-invasive tool that allows assessing spatiotemporal dynamics between the characteristics of functional brain networks and pathological changes. RsfMRI has been applied in the APP^NL-F/NL-F^ and APP^NL/NL^ mice at 3 and 7 months of age to establish whether their increased Aβ42/40 ratio and concomitant early Aβ pathology are associated with deficits in brain function. At 3 months of age, APP^NL-F/NL-F^ mice showed hypersynchrony of BOLD FC *within* the hippocampus and frontal/cingulate networks compared to APP^NL/NL^ mice. Deficits of the hippocampus could affect learning and memory abilities, but the specific early involvement of frontal brain regions could be an additional early indicator of impairments in the ability to elaborate new rules, or cognitive flexibility, which were observed in 3 months old APP^NL-F/NL-F^ mice. In line with these data, previous reports show that 5XFAD mice demonstrate early cognitive deficits related to frontal brain regions that occur before hippocampal-dependent learning impairments^23^. Additionally, 3 months old APP^NL-F/NL-F^ mice demonstrate hypersynchronized FC *between* functional brain networks compared to APP^NL/NL^ mice, more specifically involving the hippocampal, cingulate-frontal, frontal-thalamic, default-mode like, striatal (caudate putamen), and sensorimotor networks. BOLD FC was specifically increased between hippocampus and prefrontal cortex, hippocampus and retrosplenial cortex, striatum and hippocampus, and striatum and cingulate cortex. These are connections that are reportedly involved in cognitive flexibility^9^. More importantly, these data indicate changes in brain function at an early stage of pathology in APP^NL-F/NL-F^ mice, occurring before Aβ deposition.

In contrast, APP^NL-F/NL-F^ mice showed telencephalic hyposynchrony of BOLD FC at 7 months of age. This change was less extensive than the hypersynchrony observed at 3 months of age, but still involved the hippocampal, striatal and default mode-like networks. Hyposynchronous BOLD FC was observed between hippocampus and prefrontal cortex, striatum and hippocampus, and striatum and cingulate cortex. This dynamic pattern of hypersynchrony before Aβ deposition and subsequent hyposynchrony at later age is consistent with other studies in transgenic APP mouse models. We previously reported that TG2576 (APPK670/671 L Swedish) and PDAPP (APP V717F Indiana) transgenic mice display early hypersynchrony of BOLD FC in hippocampus and frontal cortex, respectively^14^. This early hypersynchrony was associated with increased levels of pre-plaque stage Aβ, and in TG2576 mice, this altered BOLD FC (and synaptic deficits) could be prevented by an anti-Aβ antibody. At later stages of Aβ deposition, both TG2576 and PDAPP transgenic mouse models displayed hyposynchronous telencephalic BOLD FC^14^, which was also reported in APP/PS1 transgenic mice at advanced stages of Aβ pathology^16^. Notably, this phasic effect on brain BOLD FC appears to be translationable to clinical AD as early hypersynchrony was reported in children carrying the PSEN1 mutation^24^. Moreover, late stage hyposynchronous telencephalic BOLD FC has been observed consistently at more advanced stages of AD pathology^12^.

Apparently, telencephalic networks in APP^NL-F/NL-F^ mice progress from a hypersynchronous state to hyposynchrony between 3 and 7 months of age. It has been shown that hypersynchrony of BOLD FC in transgenic mouse models is associated with increased ratio of excitatory/inhibitory functioning^4,14,25^, probably caused by the damaging effects of pathologically increased Aβ42/40 ratio, which is increased from 3 months of age onwards in the APP^NLF/NLF^ mice (Figure S2A). This hyper-to-hyposynchrony shift at the functional network level could be caused by progressive damage induced by this hyperexcitability and the complex neurotoxic effects of Aβ42 (note that between 3–7 months Aβ deposition is still low) (Figure S2B)^8^. More severe cognitive deficits reported in this model at advanced ages^17^ could have resulted from progressive synaptic and neural network defects.

The reversal defects we observed in APP^NL-F/NL-F^ mice could be considered to be relatively mild compared to the extensive telencephalic hypersynchrony of BOLD FC in these mice. However, it has been well established that during the preclinical phase of AD, brain network dysfunctions also occur in absence of overt cognitive symptoms^26^. Thus, FC MRI could be a useful tool to determine early stage pre-symptomatic changes in functional brain networks.

## Material and Methods

### Animals

Female APP^NL-F/NL-F^ knock-in mice (APP KM670/671 N Swedish, APP I716F Iberian) were compared to age-matched APP^NL/NL^ knock-in mice (APP KM670/671 N Swedish) at 3 months (APP^NL-F/NL-F^ N = 9, APP^NL/NL^ N = 13) and 7 months of age (APP^NL-F/NL-F^ N = 9, APP^NL/NL^ N = 10). APP knock-in mice^8^ were derived from the Riken Institute colony (Laboratory for Proteolytic Neuroscience, PI: Dr. Takaomi Saido, Riken Brain Science Institute, Japan). APP^NLF/NLF^ mice show a progressive increase of Aβ42/Aβ40 ratio at 3 months of age compared to wild-type and APP^NL/NL^ mice (Figure S2A), and the first Aβ plaques deposit around 6 months of age (Figure S2B). APP^NL/NL^ mice do not develop Aβ plaques during their entire lifespan and are considered an appropriate negative control for APP^NL-F/NL-F^ mice as the levels of APP, APP intracellular domain (AICD) and C-terminal fragment β (CTF-β) are equivalent in both models, thus facilitating interpretation of the effects of increased Aβ42/Aβ40 ratio caused by the Iberian mutation in the APP^NL-F/NL-F^ mice (Figure S2). All procedures were performed in strict accordance with the European Directive 2010/63/EU on the protection of animals used for scientific purposes. The protocols were approved by the Committee on Animal Care and Use at KU Leuven, Belgium (permit number: P073/2013) and all efforts were made to minimize animal suffering. All mice were first subjected to rsfMRI imaging, after which the behavior tasks were performed, to avoid variation in the FC data caused by functional or structural reorganization elicited during learning procedures.

### Resting-state functional MRI

#### MRI procedures

For the MRI handling procedures all mice were anesthetized with 2.5% isoflurane (IsoFlo, Abbott, Illinois, USA), which was administered in a mixture of 70% nitrogen (400 cc/min) and 30% oxygen (200 cc/min). During the rsfMRI imaging procedures, a combination of medetomidine (Domitor, Pfizer, Karlsruhe, Germany) and isoflurane was used to sedate the animals^14^. After positioning of the animal in the scanner, medetomidine was administered subcutaneously as a bolus injection (0.3 mg/kg), after which the isoflurane level was immediately decreased to 1%. Ten minutes before the rsfMRI acquisition, isoflurane was decreased to 0.5%. RsfMRI scans were consistently acquired 40 min after the bolus injection, during which the isoflurane level was kept at 0.5%. After the imaging procedures, the effects of medetomidine were counteracted by subcutaneously injecting 0.1 mg/kg atipamezole (Antisedan, Pfizer, Karlsruhe, Germany). The physiological status of all animals was monitored throughout the imaging procedure. A pressure sensitive pad (MR-compatible Small Animal Monitoring and Gating system, SA Instruments, Inc.) was used to monitor breathing rate and a rectal thermistor with feedback controlled warm air circuitry (MR-compatible Small Animal Heating System, SA Instruments, Inc.) was used to maintain body temperature at 37.0 ± 0.5 °C.

MRI procedures were performed on a 9.4 T Biospec MRI system (Bruker BioSpin, Germany) with the Paravision 5.1 software (www.bruker.com). Images were acquired using a standard Bruker cross coil set-up with a quadrature volume coil and a quadrature surface coil for mice. Three orthogonal multi-slice Turbo RARE T2-weighted images were acquired to render slice-positioning uniform (repetition time 2000 ms, echo time 33 ms, 16 slices of 0.4 mm). Field maps were acquired for each animal to assess field homogeneity, followed by local shimming, which corrects for the measured inhomogeneity in a rectangular VOI within the brain. Resting-state signals were measured using a T2*-weighted single shot EPI sequence (repetition time 2000 ms, echo time 15 ms, 16 slices of 0.4 mm with a gap of 0.1 mm, 300 repetitions). The field-of-view was (20 × 20) mm^2^ and matrix size (128 × 64), resulting in voxel dimensions of (0.156 × 0.312 × 0.5) mm³.

#### MRI data pre-processing

Pre-processing of the rsfMRI data, including realignment, normalization and smoothing, was performed using SPM8 software (Statistical Parametric Mapping, http://www.fil.ion.ucl.ac.uk). First, all images within each session were realigned to the first image. This was done using a least-squares approach and a 6-parameter (rigid body) spatial transformation. For the rsfMRI data analyses, motion parameters resulting from the realignment were included as covariates to correct for possible movement that occurred during the scanning procedure. Second, all datasets were normalized to a study specific EPI template and co-registered to an anatomical T2-weighted template. The normalization steps consisted of a global 12-parameter affine transformation followed by the estimation of the nonlinear deformations. Finally, in plane smoothing was done using a Gaussian kernel with full width at half maximum of twice the voxel size (0.31 × 0.62) mm². All rsfMRI data were filtered between 0.01–0.25 Hz using the REST toolbox (REST1.7, http://resting-fmri.sourceforge.net).

#### MRI data analysis

RsfMRI data were first analyzed with group independent component analysis (ICA) to determine which brain networks can be discerned using the GIFT-toolbox (Group ICA of fMRI toolbox version 2.0a: http://icatb.sourceforge.net/). First the data of each individual animal was concatenated. Then group ICA was performed using the Infomax algorithm, followed by back reconstruction of the data to single-subject independent components and time courses. ICA was performed using a pre-set of 15 components, which was shown to be appropriate to identify networks in mice^27,28^. Masks containing the individual brain regions resulting from the ICA analyses were defined using MRicron software (MRicron version 6.6, 2013, http://www.mccauslandcenter.sc.edu/mricro/) and used for region-of-interest (ROI) correlation analyses, where pairwise correlation coefficients between each pair of ROIs were calculated and z-transformed using an in-house program developed in MATLAB (MATLAB R2013a, The MathWorks Inc. Natick, MA, USA). Mean z-transformed FC matrices were calculated for each group. For inter-network analyses, homologous ICA components were grouped and the resulting brain networks were then used for inter-network correlation analyses. Statistical analyses of the rsfMRI data included two-sample T-tests and two-way ANOVA with Sidak correction for multiple comparisons (p < 0.05).

Additionally, seed-based analyses were performed by computing individual z-transformed FC-maps of the right hippocampus and right caudate putamen using REST toolbox, resulting in FC-maps for each of these seed regions for each group. FC between the seed-region and other regions on the FC-map were calculated by defining a mask containing the ROIs derived from the mean statistical FC-maps, and then calculating the z-values from these ROIs for each individual subject using REST-toolbox. Statistical analyses of the FC-maps included a one-sample T-test (p < 0.001, uncorrected, threshold 10 voxels) for within group analyses, and included a two-way ANOVA with Sidak correction for multiple comparisons (p < 0.05) for between group analyses of specific functional connections i.e. hippocampus bilateral, hippocampus-frontal, hippocampus-retrosplenial, caudate putamen-cingulate and caudate putamen-hippocampus.

### Spatial learning in the Morris water maze

The Morris water maze test was performed to assess spatial memory that relies on distal cues to locate a submerged platform (15 cm diameter) in an open circular swimming arena (150 cm diameter) filled with opaque water (non-toxic white paint, 26 ± 1  °C), as previously described^9^. Analyses included 10 days of acquisition training, where each daily session consisted of 4 swimming trials (15 min interval between trials) starting randomly from 4 starting positions. Swimming tracks were recorded using video hardware and Ethovision software (Noldus, The Netherlands). Mice that failed to find the platform within 120 s were guided to it and remained there for 15 s before being returned to their cages. Reference memory performance is measured as preference for the platform area when the platform is absent, and was tested during probe trials (100 s) after 5 and 10 acquisition sessions and after 5 reversal sessions, i.e. on days 6, 11, and 16 respectively. After 10 days of acquisition training, when the mice have established a robust preference for the platform location, reversal training was performed during 5 days, during which the location of the platform was changed, requiring relearning and cognitive flexibility. Analyses included calculating path length (i.e. distance traveled by the mouse before finding the platform), % time spent in the target quadrant during the 15 training sessions, and % time spent in each quadrant during the probe trials. Statistical analyses included one-way and two-way repeated measures ANOVA with Sidak correction for multiple comparisons (p < 0.05).

## Electronic supplementary material

Supplementary Figures
