# Supplementary material for: Spatial reversal learning defect coincides with hypersynchronous telencephalic BOLD functional connectivity in APPNL-F/NL-F knock-in mice
Source: Sci Rep. 2018 Apr 19;8:6264. doi: 10.1038/s41598-018-24657-9 (PMC5908850; doi:10.1038/s41598-018-24657-9)
Supplement: Supplementary file 1 — Supplementary Figures [file 41598_2018_24657_MOESM1_ESM.pdf]

Spatial reversal learning defect coincides with hypersynchronous telencephalic BOLD functional connectivity in APP$^{NL\text{-}F/NL\text{-}F}$ knock-in mice

Disha Shah[1,2,3*], Amira Latif Hernandez[2*], Bart De Strooper[3], Takashi Saito[4], Takaomi Saido[4], Marleen Verhoye[1], Rudi D'Hooge[2], Annemie Van der Linden[1]

[1] Bio-Imaging Lab, University of Antwerp, Belgium

[2] Laboratory of Biological Psychology, KU Leuven, Belgium

[3] VIB Center for the Biology of Disease, KU Leuven, Belgium

[4] Laboratory for Proteolytic Neuroscience, Riken Brain Science Institute, Japan

* Equal contribution

Corresponding authors: Annemie Van der Linden, PhD; Disha Shah

annemie.vanderlinden@uantwerpen.be; disha.shah@kuleuven.be

Supplementary Figures

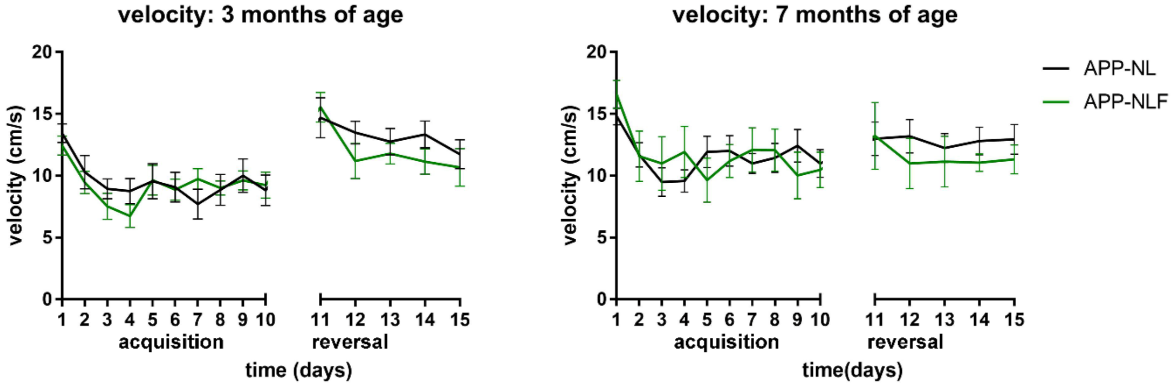

Figure S1: Velocity during the Morris water maze behaviour task. Velocity (cm/s) ± standard error shown for the acquisition and reversal phase of the Morris water maze task for 3 and 7 months old $APP^{NL/NL}$ and $APP^{NLF/NLF}$ mice. RM-ANOVA showed no significant differences between groups.

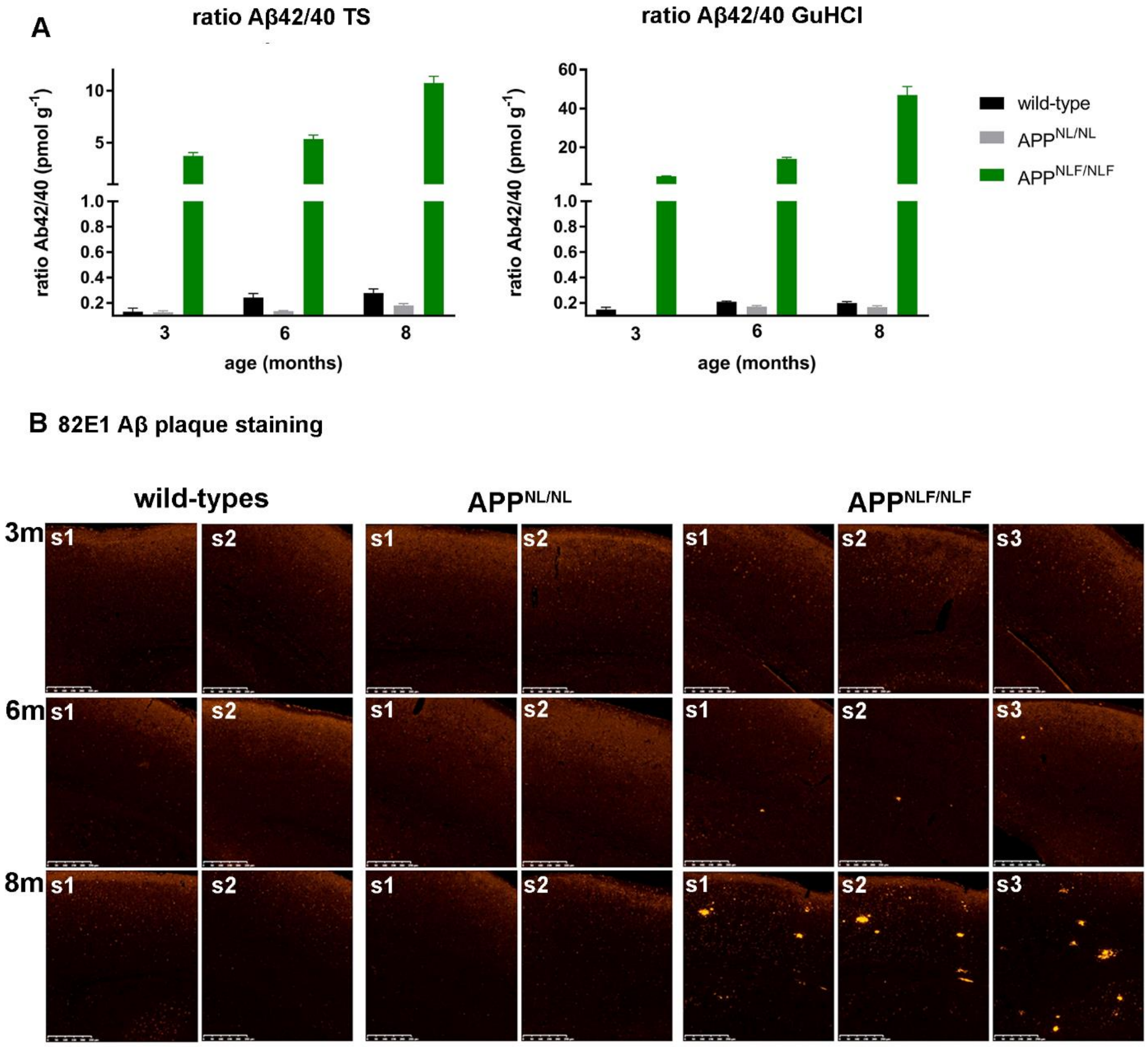

<u>Figure S2:</u> Aβ pathology. A) Biochemical quantities of Aβ42/40 ratio in the brains of wild-type, $APP^{NL/NL}$ and $APP^{NLF/NLF}$ mice at 3 (N=3/group), 6 (N=3/group) and 8 (wild-types N=3, $APP^{NL/NL}$ N=3, $APP^{NLF/NLF}$ N=4) months of age. Aβ levels (pmol/g) were measured from Tris-HCl-buffered saline (TS) and guanidine-HCl (GuHCl) fractions and quantified by sandwich ELISA as previously described[1]. Data are shown as ratio Aβ42/40 ± standard error. **B)** Brain sections from 3, 6 and 8 months old wild-type (N=2/age group), $APP^{NL/NL}$ (N=2/age group) and $APP^{NLF/NLF}$ (N=3/age group) were immunostained with 82E1 N-terminal specific Aβ antibody (IBL, Japan) to detect Aβ plaques using a previously described protocol[1]. Scale bars represent 250 µm.

References:

1. Saito, T. *et al.* Single App knock-in mouse models of Alzheimer's disease. *Nat Neurosci* **17,** 661–663 (2014).
